# An integrative approach to detecting potential blood-based biomarkers of cognitive frailty

**DOI:** 10.1016/j.jnha.2025.100726

**Published:** 2025-11-24

**Authors:** Motoki Furutani, Mutsumi Suganuma, Tohru Hosoyama, Risa Mitsumori, Marie Takemura, Yasumoto Matsui, Yukiko Nakano, Shumpei Niida, Kouichi Ozaki, Shosuke Satake, Daichi Shigemizu

**Affiliations:** a Medical Genome Center, Research Institute, National Center for Geriatrics and Gerontology, Aichi, Japan; b Department of Cardiovascular Medicine, Hiroshima University Graduate School of Biomedical and Health Sciences, Hiroshima, Japan; c Geroscience Research Center, Research Institute, National Center for Geriatrics and Gerontology, Aichi, Japan; d Center for Frailty and Locomotive Syndrome, National Center for Geriatrics and Gerontology, Aichi, Japan; e Research Institute, National Center for Geriatrics and Gerontology, Aichi, Japan; f RIKEN Center for Integrative Medical Sciences, Yokohama, Japan; g Department of Geriatric Medicine, Hospital, National Center for Geriatrics and Gerontology, Aichi, Japan; h Department of Frailty Research, Center for Gerontology and Social Science, Research Institute, National Center for Geriatrics and Gerontology, Aichi, Japan

**Keywords:** Blood-based biomarker, Cognitive frailty, Metabolome, Myristic acid, Prediction model

## Abstract

**Objective:**

Cognitive frailty, defined by the coexistence of cognitive decline and physical frailty, has been clinically defined, but its biological clues are still vague. This underscores the need for promising blood-based molecular biomarkers.

**Design:**

Cross-sectional observational study.

**Settings and participants:**

Frailty was diagnosed using the Japanese version of the Cardiovascular Health Study (J-CHS), and mild cognitive impairment was assessed with the Japanese version of the Montreal Cognitive Assessment (MoCA-J) and Mini-Mental State Examination-Japanese (MMSE-J). Participants with MMSE-J ≥24, MoCA-J score ≤25, and J-CHS score ≥1 were classified as having cognitive frailty. This study included 87 older adults aged ≥65 years, comprising 44 robust and 43 with cognitive frailty.

**Measurements:**

Blood samples and associated clinical data were obtained from the National Center for Geriatrics and Gerontology Biobank in Japan. A multi-omics analysis integrating clinical data, RNA-seq, aging-related factors, and metabolomics were conducted to identify potential biomarkers through logistic regression, adjusting for age, sex, and body mass index (BMI). An optimal set of biomarkers was determined by constructing prediction models using the random forest algorithm.

**Results:**

Three candidate biomarkers were identified from aging-related factors—growth differentiation factor (GDF15), brain-derived neurotrophic factor (BDNF), and Adiponectin—and three from metabolomics—myristic acid, nicotinamide, and γ-butyrobetaine. Using combinations of these candidates with clinical variables, we constructed risk prediction models. The best model incorporated one aging-related factors (GDF15) and two metabolites (myristic acid, and nicotinamide), achieving a high area under the receiver operating characteristic curve (AUC) of 0.96 in an independent validation cohort. This was significantly higher than models based solely on clinical information (age, sex, and BMI) (Welch’s t-test, *p* <0.001). Among these biomarkers, myristic acid showed the highest influence, with a median Gini importance of 0.38 (95% confidence interval: 0.29–0.47).

**Conclusions:**

We identified three promising biomarkers—GDF15, myristic acid, and nicotinamide—for cognitive frailty. Notably, low plasma myristic acid levels emerged as the most significant contributor to the prediction model. Further refinement and large-scale validation will be essential to support its future clinical application.

## Highlights

• Cognitive frailty, defined by the coexistence of cognitive decline and physical frailty, presents significant health risks.
• We identified three promising biomarkers: GDF15, myristic acid, and nicotinamide.
• Low plasma myristic acid levels were crucial in predicting cognitive frailty, emphasizing their potential as a biomarker.

## 1 Introduction

The global population of older adults is increasing rapidly, with the Statistics Bureau, Ministry of Internal Affairs and Communications in Japan reporting in 2024 that 29.3% of its total population was aged 65 and over. Consequently, the prevalence of frailty and dementia among older adults is also rising, presenting substantial challenges for healthcare systems and society [1]. In Japan, the prevalence of frailty among older adults over 65 years old has been reported as 11.3%, of mild cognitive impairment (MCI), 18.8%, and of coexisting frailty and MCI, 2.7%, and significant relationships between frailty and MCI have been observed [2].

Frailty is a geriatric syndrome characterized by a decline in multiple physiological and cognitive functions [3]. The Cardiovascular Health Study (CHS) criteria are widely used internationally to diagnose physical frailty [3]. Japan uses a modified version, the Japanese version (J-CHS) [4], which accounts for population-specific physical differences. Cognitive impairment is typically assessed by using the Mini-Mental State Examination-Japanese (MMSE-J) or the Montreal Cognitive Assessment (MoCA), or both [5,6]. The coexistence of cognitive decline and physical frailty, known as cognitive frailty, was defined by the International Academy of Nutrition and Aging and the International Association of Gerontology and Geriatrics (IANA/IAGG) [7] and there are many criteria for cognitive frailty includes not only frailty but also pre-frailty [8,9]. Recent studies have linked cognitive frailty to an increased mortality risk [10]. Numerous independent studies have investigated physical frailty [3,11] and cognitive impairment [12,13]. However, although blood biomarkers are emerging as promising tools for diagnosing geriatric diseases [14,15], an effective method for diagnosing cognitive frailty has yet to be established.

Several frailty biomarkers have been identified in relation to key biological processes or features, including inflammation, mitochondrial function and apoptosis, calcium homeostasis, fibrosis, neuromuscular junctions, neurons, and the cytoskeleton and hormones [16,17]. Similarly, biomarkers associated with cognitive impairment—particularly those linked to neurodegeneration—have been reported [16,18]. RNA-sequencing (RNA-seq) analysis of human peripheral blood mononuclear cells has revealed blood-based biomarkers of Alzheimer’s disease [15]. Recently, metabolomic analyses have identified blood-based biomarkers, such as Creatine and urine diphosphate acetyl-carnosine, that are specifically associated with frailty and cognitive decline [19].

Although numerous studies have explored biomarkers for frailty, few have focused specifically on cognitive frailty, and even fewer have conducted multi-omics analyses. Frailty and cognitive decline are inevitable processes of aging, suggesting the existence of underlying biological mechanisms. With this in mind, to identify potential biomarkers of cognitive frailty, we performed a multi-omics analysis—including clinical data, RNA-seq data, aging-related factors, and metabolome data—by using biobank blood samples from older adults whose physical and mental functions had been assessed by medical professionals. We identified three candidate biomarkers of cognitive frailty from the aging-related factors analysis and three from the metabolome analysis. By combining these biomarkers with clinical information, we developed an optimal risk-prediction model that achieved strong performance, as reflected by a high area under the curve (AUC) in an independent validation cohort. In particular, all the identified biomarkers showed significant correlations with the J-CHS frailty diagnostic criteria components. These findings suggest that the biomarkers we identified could play a crucial role in the clinical diagnosis of cognitive frailty and, ultimately, could help to improve the healthy life expectancy of older adults.

## 2 Methods

### 2.1 Sample collection

The National Center for Geriatrics and Gerontology (NCGG) established the Locomotor Frailty Sarcopenia Registry to collect comprehensive evidence on the locomotive syndrome, frailty, and sarcopenia. A toral of 850 older adults were enrolled, with their blood samples and clinical information stored in the NCGG biobank.

Frail older adults were diagnosed according to the J-CHS criteria [4], which consist of the following five components: shrinking (unintentional weight loss of ≥2 kg in the past 6 months, “Yes” = 1 point), exhaustion (feeling tired without a reason in the past 2 weeks, “Yes” = 1 point), low activity (engagement in only moderate or low levels of physical exercise or sports aimed at health, “No” = 1 point), slowness (gait speed <1.0 m/s, 1 point), and weakness (grip strength <28 kg in males or <18 kg in females, 1 point). The MMSE-J was used to assess cognitive function in older adults. Those with suspected dementia (with an MMSE-J score ≤23) were excluded from the study. The Japanese version of the MoCA (MoCA-J) was used to screen for MCI. Participants with a MOCA-J score ≤25 and a J-CHS score ≥1 were categorized as having cognitive frailty. The participants with J-CHS score = 0, MMSE-J ≥28 and, MoCA-J >25 were classified as robust.

**Fig. 1:**
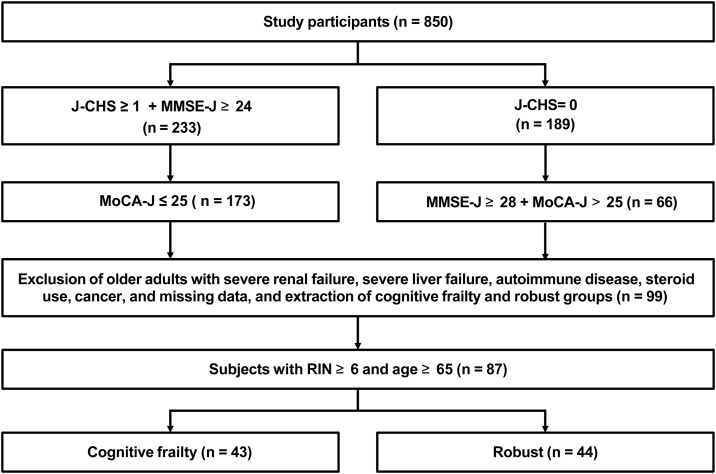
Study flowchart. Abbreviation: J-CHS = Japanese version of the Cardiovascular Health Study; MoCA-J, Japanese version of the Montreal Cognitive Assessment; MMSE-J = Mini-Mental State Examination-Japanese; RIN = RNA integrity number.

Of the 850 participants, 173 were classified as having cognitive frailty, whereas 66 were classified as robust [4,[7], [8], [9]]. Of them, older adults with severe renal failure, severe liver failure, autoimmune disease, steroid users, cancer were excluded. Finally, 87 high-quality samples, with an RNA integrity number (RIN) ≥6 and from older adults aged ≥65 years, were used to construct the sequencing library. These samples were from 43 cognitive frailty older adults and 44 robust older adults (Fig. 1).

### 2.2 Clinical data analysis

A total of 58 clinical variables were examined, measured in at least 77 of the 87 older adults. They consisted of six physical performance measures (skeletal muscle mass index [SMI], systolic blood pressure, diastolic blood pressure, heart rate, young adult mean [YAM] of the lumbar spine, and YAM of the femoral neck), number of years of education, and 51 routine blood test items (Supplementary Table 1).

SMI was measured using the InBody device, which employs a multi-frequency bioelectrical impedance analysis method and measures frequencies ranging from 1 to 3000 kHz. Blood pressure was measured twice using a sphygmomanometer after participants had been seated and rested for 5 min. The lower of the two measurements for systolic blood pressure and diastolic blood pressure was used for analysis. Heart rate was recorded simultaneously with blood pressure. Bone mineral density was measured at the lumbar spine (L2 to L4) and femoral neck by using dual-energy X-ray absorptiometry (DEXA) and obtained YAM of both femoral neck and lumbar spine (L2 to L4). Blood samples were centrifuged at the examination sites and stored at −80 °C until analysis.

The associations of clinical items between cognitive frailty and robust were assessed using logistic regression, adjusted for age, sex, and body mass index (BMI). The analysis was performed using the Python package statsmodels (version 0.13.2). Statistical significance was determined using the false discovery rate (FDR) method, with a threshold of <0.05.

### 2.3 RNA-seq data analysis

All RNA-seq data were downloaded from the NCGG Biobank database [15]. The method of RNA-seq data analysis was summarized in Supplementary Method 1. Differentially expressed genes (DEGs) were defined as those with normalized transcripts per million (nTPM) ≥1 in total peripheral blood mononuclear cells (PBMCs), based on the Human Protein Atlas (https://www.proteinatlas.org), with |log2 fold change (FC)| >1 and FDR <0.05, in order to exclude lowly expressed genes [[20], [21], [22], [23]]. FDR values were calculated by using the Benjamini–Hochberg method.

### 2.4 Enzyme-linked immunosorbent assay

A total of 10 humoral factors were reported as possible biomarkers of age-related frailty [16]; namely, tumor necrosis factor alpha (TNFα, R&D Systems, Minneapolis, MN), growth differentiation factor 15 (GDF15, R&D Systems), chemokine (C-X-C motif) ligand 9 (CXCL9, R&D Systems), CXCL10 (R&D Systems), fibroblast growth factor 21 (FGF21, R&D Systems), apelin (Apelin-12; Phoenix Pharmaceuticals, Burlingame, CA), progranulin (AdipoGen Life Sciences, San Diego, CA), brain-derived neurotrophic factor (BDNF, Fujifilm-Wako, Osaka), extracellular nicotinamide phosphoribosyltransferase (eNAMPT, AdipoGen Life Science), and adiponectin (total Adiponectin; R&D Systems), were measured by using the respective enzyme-linked immunosorbent assays (ELISAs) in accordance with the manufacturers’ protocols. Whole blood samples were collected from the NCGG biobank, and serum blood samples were centrifuged at 3000 rpm for 10 min and the supernatants were collected. Duplicate measurements were performed for all assays, and the average value of the two measurements was used for data analysis. If necessary, samples were diluted with the buffers in the ELISA kits before the assay.

The associations of the possible biomarkers of age-related frailty between cognitive frailty and robust samples were assessed by logistic regression, adjusting for age, sex, and BMI, by using the Python package statsmodels (ver. 0.13.2). An FDR <0.05 was considered statistically significant.

### 2.5 Metabolome analysis

All samples were analyzed by using a capillary electrophoresis–time-of-flight mass spectrometry (CE-TOFMS) system (Agilent Technologies, Santa Clara, CA) in collaboration with Human Metabolome Technologies, Inc. (Yamagata, Japan). Both cationic and anionic metabolites were profiled through CE-TOFMS-based metabolome analysis. Peaks detected by CE-TOFMS were processed by using the automatic integration software MasterHands ver.2.19.0.2 (Keio University, Tsuruoka, Japan). Metabolites were identified and quantified by comparing reference peak information with standards, including m/z, migration time, and peak area. Peak areas were normalized to internal standard levels and adjusted for sample amounts to calculate relative metabolite levels. Relative values were obtained for 199 metabolites, whereas quantitative values were obtained for 53 metabolites. Metabolites with missing values in 10 or more individuals were excluded, resulting in a final dataset of 84 relative and 44 quantitative metabolite values.

### 2.6 Risk-prediction model construction

An outline of our risk-prediction model construction is presented in Supplementary Fig. 1. Two-thirds of the collected data (n = 87) were used as a discovery data set (n = 65; cognitive frailty = 32; robust = 33), and the remaining one-third were used as a validation data set (n = 22; cognitive frailty = 11; robust = 11). The discovery data set was used to select candidate biomarkers during each cross-validation step. Candidate biomarkers were identified through analyses of clinical data, ELISA data, metabolome data, and RNA-seq data. Risk-prediction models were constructed using combinations of the candidate biomarkers with clinical information (age, sex, and BMI) and a random forest classifier. A five-fold cross-validation was employed to determine the optimal candidate biomarkers within the discovery data set. The final model was constructed using the entire discovery data set, and its performance was evaluated on the validation data set by using the AUC of the receiver operating characteristic curve. All analyses and their visualizations were implemented by using the Python packages scikit-learn (ver. 1.0.2), matplotlib (ver. 3.5.1), and seaborn (ver. 0.11.2).

### 2.7 Relationship between candidate biomarkers, J-CHS frailty criteria and cognitive function

We performed a logistic regression to examine the relationship between the candidate biomarkers and the J-CHS frailty criteria, and linear regression to examine the relationship between MMSE-J, MoCA-J and the candidate biomarkers. We performed association study adjusted for age, sex and BMI. We also estimated correlation (Pearson correlation coefficient) between candidate biomarkers, J-CHS frailty criteria and cognitive function. All analyses and their visualizations were implemented by using the Python packages statsmodels (version 0.13.2), scipy (version 1.7.3), matplotlib (ver. 3.5.1), and seaborn (ver. 0.11.2).

## 3 Results

### 3.1 Demographic data of study participants

The demographic data of the 87 older adults (robust = 44, cognitive frailty = 43) enrolled in the study are summarized in Table 1. Whereas no statistically significant differences in age, sex, or BMI were observed between phenotypes, significant differences were found for each individual J-CHS component by using Fisher’s exact test (*p* <0.05). Among clinical variables, there were significant differences were observed in the education years, Kihon Checklist (A tool for adults aged 65 and older to assess daily living, health status, and functional decline in Japan [24]), serum albumin, and C-reactive protein (Table 1). The distribution of frailty scores among the 43 cognitive frailty older adults indicated a high prevalence of slowness, followed by low activity, then exhaustion and weakness, and finally shrinking (Supplementary Fig. 1). There were no older adults with a J-CHS of 1 in cognitive frailty.

**Table 1:** Demographic data of the study participants.

Variable, mean ± SD	Robust (n = 44)	Cognitive frailty (n = 43)	*p* value
Age (y)	74.32 ± 5.43	76.35 ± 7.47	0.12^a^
Sex, n (%)			0.47^b^
Males	10 (22.73%)	13 (30.23%)	
Females	34 (77.27%)	30 (69.77%)	
BMI (kg/m^2^)	23.68 ± 2.16	25.57 ± 5.27	0.080^a^
Total J-CHS score	0	2.51 ± 0.63	2.33E-18^a^, *
Shrinking, n (%)	0	7 (16.28%)	5.51E-03^b^, *
Exhaustion, n (%)	0	14 (32.56%)	1.45E-05^b^, *
Low activity, n (%)	0	30 (69.77%)	1.87E-13^b^, *
Slowness, n (%)	0	43 (100.00%)	7.62E-26^b^, *
Weakness, n (%)	0	14 (32.56%)	1.45E-05^b^, *
Clinical Variables			
Hypertension, n (%)	39 (88.63%)	34 (79.07%)	0.26^b^
CVD, n (%)	10 (22.72%)	8 (18.60%)	0.79^b^
Education years	12.50 ± 2.65	10.90 ± 2.47	0.0041^a^, *
Kihon Checklist	3.43 ± 2.27	8.21 ± 3.64	1.70E-9^a,^^a^
WBC ($\times 10^{2}$/ul)	52.70 ± 11.50	56.30 ± 14.80	0.28^a^
Hemoglobin (g/dl)	13.20 ± 1.08	12.60 ± 1.49	0.099^a^
Albumin (g/dl)	4.26 ± 0.25	4.07 ± 0.32	0.044^a^, *
AST (U/L)	21.50 ± 5.74	23.30 ± 9.64	0.88^a^
ALT (U/L)	18.40 ± 6.51	19.40 ± 9.21	0.87^a^
Creatinine (mg/dl)	0.71 ± 0.17	0.70 ± 0.18	0.94^a^
CRP (mg/dl)	0.11 ± 0.23	0.17 ± 0.30	0.0034^a^, *

Abbreviation: SD, standard deviation; MCI, mild cognitive impairment;

J-CHS, The Japanese version of the Cardiovascular Health Study;

CVD, cardiovascular disease (includes coronary artery disease, peripheral artery disease, arrhythmia, and heart failure);

WBC, white blood cells;

AST, Aspartate aminotransferase; ALT, alanine aminotransferase;

HbA1c, Hemoglobin A1c; CRP, C-reactive protein.

a Mann–Whitney U test.

b Fisher’s exact test.

* *p* < 0.05.

### 3.2 Clinical data analysis

We examined 58 clinical items that are not currently used to diagnose frailty. A logistic regression model, adjusted for age, sex, and BMI, was applied to assess differences between the cognitive frailty and robust phenotypes for each clinical item. No statistically significant differences were found between the two phenotypes for any of the clinical items (Supplementary Table 1).

### 3.3 RNA-seq data analysis

RNA-seq analysis was performed on 87 blood samples from subjects with cognitive frailty and robust older adults (Supplementary Table 2). An average of 26.2 million raw read sequences were obtained from the cognitive frailty subjects and 30.5 million from the robust subjects; 96.1% and 98.6% were high-quality (i.e., >Q20) read sequences, respectively. After we discarded the low-quality read sequences and trimmed reads with adaptor sequences, >23.8 million reads of cleaned data remained. Of these, >81.2% were uniquely mapped to a human reference genome (GRCh37) across the two phenotype groups. We examined the DEGs between cognitive frailty and robust subjects. A total of 18,545 genes with count per million (CPM) >1 in more than 25% of all sequenced samples were analyzed. No significant DEGs that satisfied the following criteria (FDR <0.05, |log₂ FC| >1, and nTPM ≥1 in total PBMCs from the Human Protein Atlas) were found (Supplementary Figure 3).

### 3.4 ELISA analysis

We measured 10 humoral factors reported as possible biomarkers of age-related frailty (TNFα, GDF15, CXCL9, CXCL10, FGF21, apelin, progranulin, BDNF, eNAMPT, and adiponectin) by using ELISA. A logistic regression model, adjusted for age, sex, and BMI, was employed to assess the associations between cognitive frailty and robust older adults. Three of these factors—GDF15, BDNF, and adiponectin—showed significant associations with cognitive frailty at an FDR threshold of <0.05. All three exhibited higher levels in individuals with cognitive frailty than in robust individuals. Among them, GDF15 had the highest association with cognitive frailty (Odds ratio [OR] = 6.55, 95% CI [confidence interval] = 2.03–21.1, FDR <0.05, Table 2).

**Table 2:** Results of age-related factor analysis.

Age-related factor	Robust Mean ± SD	Cognitive frailty Mean ± SD	Odds ratio (95%CI)	*p* value	FDR
**GDF15 (ng/mL)**	1.1 ± 0.27	1.59 ± 0.71	6.55 (2.03−21.1)	1.66E-03	**0.017**
**BDNF (pg/mlL)**	13.2 ± 3.44	15.3 ± 3.98	2.11 (1.16-−3.84)	0.014	**0.047**
**Adiponectin (ug/mlL)**	10.2 ± 4.46	14.5 ± 8.51	2.28 (1.19-−4.39)	0.013	**0.047**
Progranulin (ng/mL)	166 ± 31.1	182 ± 37.9	1.67 (0.94−2.98)	0.082	0.21
TNFa (pg/mlL)	1.54 ± 2.35	3.13 ± 5.1	1.46 (0.76-−2.81)	0.26	0.29
FGF21 (pg/mlL)	186 ± 108	227 ± 122	1.47 (0.82−2.61)	0.19	0.29
CXCL9 (pg/mL)	230 ± 147	324 ± 260	1.58 (0.75−3.34)	0.23	0.29
Apelin (ng/mL)	105 ± 25.4	112 ± 24.6	1.39 (0.81−2.38)	0.24	0.29
eNAMPT (ng/mlL)	1.15 ± 0.51	1.47 ± 0.77	1.62 (0.82-−3.21)	0.17	0.29
CXCL10 (pg/mL)	92.2 ± 74.2	117 ± 127	1.00 (0.52−1.89)	0.99	0.99

Abbreviation: SD, standard deviation; CI, confidence interval; FDR, false discovery rate;

GDF15, growth differentiation factor 15; BDNF, brain-derived neurotrophic factor;

TNFα, tumor necrosis factor alpha; FGF21, fibroblast growth factor 21;

CXCL9, chemokine (C-X-C motif) ligand 9;

eNAMPT, extracellular nicotinamide phosphoribosyltransferase;

CXCL10, chemokine ligand 10.

Significant associations with an FDR <0.05 are shown in bold font.

### 3.5 Metabolome analysis

A total of 128 metabolites were evaluated for their associations with cognitive frailty or robustness by using a logistic regression model, adjusting for age, sex, and BMI (Supplementary Table 3). Of them, four metabolites—myristic acid, 4-methylpyrazole, nicotinamide, and γ-butyrobetaine—showed significant associations with cognitive frailty at an FDR threshold of <0.05. Levels of all four were lower in older adults with cognitive frailty than in robust older adults. Among them, 4-methylpyrazole is known as the competitive inhibitor of human alcohol dehydrogenase [25] and protects against acetaminophen-induced hepatoxicity by preventing mitochondrial dysfunction and the formation of reactive oxidant species [26]. However, there are no reliable reports that 4-methylpyrazole occurs as a natural endogenous metabolite or a common food constituent. The Human Metabolome Database (HMDB) classifies it as “exogenous”, and regulatory/clinical drug sources list it solely as a pharmaceutical agent [27] (https://www.hmdb.ca/metabolites/HMDB0015344). Accordingly, we considered the detection of 4-methylpyrazole to be likely exogenous or artifactual and therefore excluded it from further analysis. Among three candidates, myristic acid had the strongest association with cognitive frailty (OR = 0.18, 95% CI = 0.074–0.43, FDR <0.05, Table 3).

**Table 3:** Results of metabolome analysis.

Metabolite (uM)	Robust Mean ± SD	Cognitive frailty Mean ± SD	Odds ratio* (95% CI)	*p* value	FDR
Myristic acid	0.042 ± 0.0096	0.027 ± 0.010	0.18 (0.074–0.43)	1.16E-04	0.015
Nicotinamide	0.031 ± 0.010	0.022 ± 0.0085	0.30(0.14–0.62)	1.28E-03	0.047
γ-Butyrobetaine	0.024 ± 0.0045	0.021 ± 0.0051	0.33 (0.17–0.65)	1.46E-03	0.047

Abbreviation: SD, standard deviation; CI, confidence interval; FDR, false discovery rate.

* Logistic regression adjusted for age, sex and body mass index.

### 3.6 Construction of risk-prediction model

**Fig. 2:**
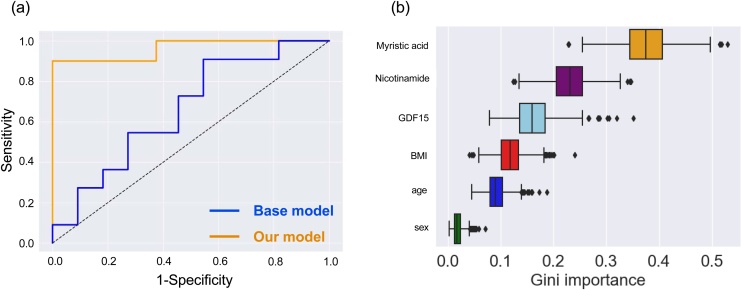
Construction of risk-prediction models. (a) Receiver operating characteristic curves of models constructed by using only basic clinical information (age, sex, and body mass index) or by using basic clinical information plus three potential biomarkers. (b) Gini importance of the model constructed by using basic clinical information plus five potential biomarkers. Data are represented as box and whisker plots depicting minimum, lower quartile, median, upper quartile, and maximum values. Abbreviation: BMI = body mass index; GDF15 = Growth differentiation factor 15.

A total of six biomarker candidates were identified from the ELISA analysis (GDF15, BDNF, and adiponectin) and the metabolome analysis (myristic acid, nicotinamide, and γ-butyrobetaine). We constructed risk-prediction models using a combination of the six candidates and three clinical factors (age, sex, and BMI) with a random forest classifier. Two-thirds of all samples were used for a discovery set and one-third for a validation set. The optimal combination of the biomarker candidates was determined in the discovery set by using five-fold cross-validation. The final model was constructed on the basis of the entire discovery set, and the adjusted model was evaluated on an independent validation set by using the AUC. The best model achieved an AUC of 0.96 (sensitivity = 0.90; specificity = 0.88) using the validation data set when three biomarkers (GDF15, myristic acid, and nicotinamide) were used. We also used a bootstrap strategy with 1000 re-samplings and estimated the 95% CI of the AUC, the sensitivity, and the specificity (AUC, 0.87–1.00; sensitivity 0.69–1.00; specificity 0.67–1.00; Fig. 2).

To estimate the effectiveness of the three biomarkers, we compared our prediction model with one constructed by using only clinical information; the latter achieved a lower AUC of 0.63 (95% CI 0.46–0.78). Risk-prediction models with the three biomarkers and the clinical information gave significantly higher AUCs than those constructed with only clinical information (Welch’s t-test *p* <0.001). Among the three biomarkers, myristic acid was the most significant contributor to the prediction model, with a median Gini importance of 0.38 (95% CI 0.29–0.47). The other biomarkers had the following median Gini importances: nicotinamide (0.23, 95% CI: 0.16−0.31); GDF15 (0.16, 95% CI 0.10–0.24); BMI (0.12, 95% CI 0.072–0.17); age (0.092, 95% CI 0.063–0.14); and sex (0.019, 95% CI 0.0070–0.043) (Fig. 2).

### 3.7 Relationship between candidate biomarkers, J-CHS frailty criteria and cognitive function

We used a logistic regression model to examine the relationship between the three biomarkers detected and the J-CHS frailty criteria, and linear regression for MMSE-J and MoCA-J. Myristic acid had the greatest associations with the J-CHS components shrinking, exhaustion, low activity, slowness and weakness; GDF15 had the associations with shrinking, slowness, and weakness; and nicotinamide was associated with slowness and low activity (Supplementary Table 4). All of the biomarkers were significantly associated with MMSE-J and MoCA-J (Supplementary Table 4). Myristic acid exhibited the strongest association with the J-CHS scores and contributed significantly to the prediction model, highlighting its potential as the most promising biomarker of cognitive frailty.

We also estimated correlations (Pearson correlation coefficients) among candidate biomarkers, J-CHS frailty criteria, and cognitive function. Myristic acid was associated with all components of J-CHS frailty score (Supplementary Figure 4) and showed the strongest correlation with the overall frailty score (ρ = −0.66), shrinking (ρ = −0.34), exhaustion (ρ = −0.39), low activity (ρ = −0.44), and slowness (ρ = −0.65) among three candidate biomarkers. Nicotinamide showed the strongest correlation with MoCA-J (ρ = 0.56), while GDF15 showed the strongest correlations with weakness (ρ = 0.40) and MMSE-J (ρ = −0.44). Significant associations were also observed among the candidate biomarkers, including myristic acid–nicotinamide (ρ = 0.41), myristic acid–GDF15 (ρ = −0.28), and nicotinamide–GDF15 (ρ = −0.36) (Supplementary Figure 4).

## 4 Discussion

Age-associated declines and diseases present substantial social challenges in developed countries [1]. Over the past decade, the concept of cognitive frailty—characterized by the coexistence of physical frailty and cognitive impairment as a heterogeneous clinical condition—has gained increasing recognition [7]. Studies have shown that older adults with cognitive frailty have a higher risk of physical function decline and dementia, with these conditions mutually exacerbating each other [[28], [29], [30]]. However, the multiple definitions of cognitive frailty make its interpretation difficult. There are several diagnostic criteria [[7], [8], [9]], with the most widely used being the IANA/IAGG definition [7], which defines cognitive frailty as the coexistence of cognitive impairment and physical frailty. However, a previous study reported that the prevalence of cognitive frailty using these criteria is low (1.0–1.8%) in community-dwelling populations [31], suggesting limited clinical utility. To address this limitation, alternative models such as reversible or potentially reversible cognitive frailty, as well as the physio-cognitive decline syndrome (PCDS) proposed at the Fifth Asian Conference of Frailty and Sarcopenia in 2019 [9], which incorporates physical pre-frailty, have been suggested [32]. In this investigation, to develop blood biomarkers for early intervention and diagnosis of cognitive frailty, we analyzed a population defined as J-CHS ≥1, which included pre-frail individuals, as the cognitive frail.

To identify key factors for the early diagnosis and treatment of cognitive frailty, we explored biomarkers through an integrated analysis of metabolic, clinical, RNA-seq, and age-relating frailty data. We identified three potential biomarkers: myristic acid, nicotinamide, and GDF15. One of these biomarkers—GDF15—were detected from age-relating frailty data and are associated with hallmarks of aging [16]. GDF15 (Growth differentiation factor 15) is known as a myomitokine which is multifunctional cytokine that is broadly secreted in response to stress and tissue injury and activate GFRAL (GDNF family alpha like) receptor which linked to stress and apoptosis [16]. GDF15 has been proposed as a biomarker of age-related health [33] and is associated with cognitive performance in middle-aged adults [34]. In our investigation, GDF15 was associated with slowness, shrinking, and weakness in the J-CHS score, with the strongest correlation observed for weakness among the three candidate biomarkers. Taken together, our results suggest that GDF15 may be especially associated with skeletal muscle mass (shrinking) and function (weakness). In healthy humans, circulating levels of GDF15 transiently increase following aerobic and resistance exercise, with particularly intense and prolonged exercise eliciting elevations comparable to those observed in disease states [35,36]. These elevations typically return to baseline within 24 h [35,37]. Although the liver, heart, and skeletal muscle have been suggested as potential sources, the primary tissue of origin and local actions of GDF15 during exercise remain unclear [35]. The GDF15–GFRAL axis may contribute to exercise aversion and metabolic regulation through activation of the hypothalamic–pituitary–adrenal stress axis; however, its physiological significance has yet to be determined [38]. In contrast, in sarcopenia and mitochondrial myopathy, circulating GDF15 levels remain chronically elevated and correlate with reduced muscle mass and strength. Notably, in older adults with sarcopenia and in critically ill older adults, inverse associations between GDF15 and quadriceps muscle mass or exercise capacity have been reported [39]. Experimental overexpression of GDF15 induces muscle atrophy [39], suggesting that GDF15 may contribute to disease progression. Furthermore, an inverse relationship between serum GDF15 levels and brain white matter integrity has been reported [40]. Elevated GDF15 in cognitive frailty may indicate chronic elevation, similar to sarcopenia or mitochondrial myopathy, and may be associated with reduced muscle mass and decreased brain white matter.

The remaining two—myristic acid, and nicotinamide—were detected by metabolome analysis. Myristic acid is a saturated fatty acid found primarily in cardamom oil, coconut oil, palm kernel oil, and bovine milk [41,42]. It is considered an important factor in aging [43]. Previous studies utilizing Alyssum homolocarpum seed oil (AHSO)—which contains alpha-linolenic acid (ALA), stearic acid, β-sitosterol, and myristic acid—have demonstrated enhanced proliferation and differentiation of neural stem cells (NSCs). Among these components, myristic acid has been identified as a key driver of neurogenesis, acting not only by promoting the conversion of ALA to docosahexaenoic acid (DHA) but also by directly stimulating NSC activity [44]. The study using naturally aged mice, showed that myristic acid alleviated hippocampal aging through correcting the imbalance of GABARB2 and GABA2 expression, and suggested that myristic acid has anti-aging effect meditating GABAergic signaling. The study using naturally aged mice showed that myristic acid alleviated hippocampal aging by correcting the imbalance of GABARB2 and GABA2 expression, and suggested that myristic acid exerts anti-aging effects by modulating GABAergic signaling [45]. Additionally, myristic acid has been identified as a potential blood-based biomarker of cognitive impairment [46]. The decreased plasma levels of myristic acid observed here further support these findings. In the context of skeletal muscle, myristic acid has been shown to enhance glucose uptake in myotubes and improve skeletal muscle mass [47]. This study demonstrated that myristic acid selectively increases β-tubulin protein levels in a DGKδ-dependent manner (DGKδ is an enzyme abundantly expressed in skeletal muscle and plays an important role in the regulation of blood glucose levels) in C2C12 myotubes [47,48]. The authors further reported that myristic acid may act as a nutritional factor capable of preventing sarcopenia by ameliorating hyperglycemia through enhancing cellular glucose uptake and promoting muscle mass gain [47]. Furthermore, myristic acid is not only a biomarker of cognitive frailty but also a potential therapeutic agent. Kawano et al. reported that Camembert cheese, which is rich in myristamide (a fatty amide of myristic acid), can improve cognitive function [49]. Myristic acid showed the highest Gini importance and the strongest associations and correlations in our investigation. These findings suggest its potential as a biomarker for early diagnosis and as a therapeutic target for cognitive frailty.

Nicotinamide is a key precursor in the nicotinamide adenine dinucleotide (NAD^+^) salvage pathway, where it is recycled by nicotinamide phosphoribosyltransferase (NAMPT) into nicotinamide mononucleotide (NMN) and subsequently converted to NAD^+^, thereby maintaining cellular NAD^+^ levels essential for energy metabolism and sirtuin activity [50,51]. Aging is associated with reduced NAMPT activity and increased NAD^+^ consumption via CD38, leading to diminished NAD^+^ pools and impaired mitochondrial function [52]. Because NAD^+^ is required for sirtuin activation, its decline suppresses mitochondrial biogenesis and energy metabolism, ultimately contributing to muscle weakness and sarcopenia [51,53]. Consistently, lower nicotinamide levels observed in cognitive frailty may reflect reduced precursor availability and salvage efficiency, resulting in NAD^+^ deficiency, sirtuin inactivation, mitochondrial dysfunction, and muscle decline. These findings support the hypothesis that NAD^+^ metabolism plays an important role in the pathophysiology of muscle decline (in our study, nicotinamide was associated with frailty score components of slowness and low activity) and highlight it as a potential therapeutic target [54]. In the context of cognition, oral nicotinamide has been shown to prevent cognitive deficits in an Alzheimer’s disease (AD) mouse model and to improve memory in non-transgenic mice [55]. These effects were linked to reduced Tau protein phosphorylated at threonine 231 (Thr231-phospho-tau), increased acetylated α-tubulin, and MAP2c upregulation, indicating enhanced microtubule stability [55,56]. By modulating tau pathology and cytoskeletal dynamics, nicotinamide emerges as a promising therapeutic candidate for AD and other tauopathies [55].

We identified three candidate biomarkers (GDF15, myristic acid, and nicotinamide). All of these candidate biomarkers significantly associated with MMSE-J and MoCA-J. Among them, GDF15 and nicotinamide are likely associated with skeletal muscle mass and function through pathways involving mitochondrial dysfunction, stress responses, and impaired energy metabolism. Myristic acid appears to contribute to muscle health by enhancing skeletal muscle mass via DGKδ-dependent regulation of β-tubulin expression. Future studies are warranted to determine whether these biomarkers can serve as reliable indicators for the diagnosis of cognitive frailty.

Our study had several limitations. First, our study is limited by the relatively small sample size. We did not identify candidate biomarkers from the RNA-seq data, probably because of the small sample size. Therefore, the RNA-seq analysis might need to be repeated with more samples. In addition, the risk of overfitting in our prediction model is largely attributable to the small validation cohort (n = 22), which may have contributed to model instability. The high AUC of 0.96 (95% CI 0.87–1.00) likely reflects this limitation and highlights uncertainty of the estimate. Therefore, our findings should be interpreted with caution, and larger external validation studies will be required to confirm the robustness and generalizability of the results. Moreover, we did not account for potential confounders such as diet and medication use. As both factors can significantly influence metabolomic and transcriptomic profiles, their omission may have impacted the observed associations.

Second, our study may have been affected by selection bias, as all older adults diagnosed with cognitive frailty in our cohort exhibited slowness (gait speed <1.0 m/s). Gait speed is known as the “sixth vital sign” because it is a core indicator of health and function in aging-related diseases [[57], [58], [59], [60]]. Slow gait is also recognized as an indicator of cognitive function [61,62] and an early marker for dementia [63]. These reports support that slowness is useful for the early diagnosis of aging-related conditions such as frailty and cognitive decline, however, the possibility of selection bias cannot be excluded, and the significant associations between candidate biomarkers and slowness should be interpreted with caution. Previous longitudinal studies of frailty progression have shown that exhaustion tends to appear first, followed by slowness, low physical activity, and weakness, whereas weight loss typically emerges at a later stage closer to frailty onset [58]. In our study, slowness was observed in all older adults with cognitive frailty, suggesting that our cohort may have included older adults with relatively advanced frailty, or alternatively, that slowness may represent a particularly important component of cognitive frailty. Further investigation is warranted to clarify these possibilities. This limitation may restrict the generalizability of our findings, and validation in larger populations that also include older adults without slow gait will be required.

Third, we did not assess whether our findings could be used to evaluate the severity of cognitive frailty. In the future, we will recruit many older adults with cognitive frailty to investigate the applicability of our findings. Since both factors can significantly influence metabolomic and transcriptomic profiles, their omission may have impacted the observed associations. Finally, although our findings were derived from a cross-sectional study, they might be applicable to prospective prediction models. We will explore this possibility through a longitudinal study. Despite these limitations, our approach provides new possibilities for developing a clinical biomarker of cognitive frailty. We believe that our risk-prediction model can be applied effectively using blood-based biomarkers.

## 5 Conclusion

We identified three potential blood-based biomarkers of cognitive frailty through analyses of clinical data, RNA-seq data, aging-related data, and metabolome data. The risk-prediction model constructed with these biomarkers demonstrated a high AUC in an independent validation cohort, suggesting its effectiveness in predicting cognitive frailty. In particular, the plasma level of myristic acid contributed significantly to the prediction model and strongly correlated with the J-CHS score, highlighting its potential as a key biomarker of cognitive frailty. We believe that further refinement and validation of these biomarkers will pave the way for their future clinical application in diagnosing cognitive frailty.

## CRediT authorship contribution statement

M.F. and M.S. developed the method and performed the analyses; R.M., T.H., S.S. and Y.N. provided technical assistance; M.T., Y.M., S.N and K.O. contributed to data acquisition and the analyses; M.F., M.S. and D.S. wrote the manuscript; and S.S. and D.S. organized this work. All authors contributed to and approved the final manuscript.

## Ethics approval and consent to participant

The study protocol was approved by the ethics committee of the NCGG (approval number 1618), and the study followed the guidelines of the Helsinki Declaration. The design and performance of the study, which involved human subjects, are clearly described in the NCGG research protocol. All participation in the NCGG Biobank was voluntary, and before registering with the NCGG Biobank, all donors gave informed consent in writing for collection of the data.

## Consent for publication

Not applicable.

## Funding sources

This study was supported by grants from the 10.13039/100009619Japan Agency for Medical Research and Development (grant number JP22dk0110046 to T.H., S.S., Y.M., and D.S.); the Japan Health Research Promotion Bureau (grant number 2021-B-01 to T.H., S.S., Y.M., and D.S.); and Research Funding for Longevity Sciences from the 10.13039/501100007312National Center for Geriatrics and Gerontology (21–44 to T.H., 21−19 to S.S., and 21–24 and 23−7 to D.S.).

## Data availability

The data supporting this study's findings are not publicly available for privacy reasons, but they are available from the corresponding author upon reasonable request.

## Declaration of competing interests

The authors declare that they have no known competing financial interests or personal relationships that could have appeared to influence the work reported in this paper.
