# Supplementary material for: An integrative approach to detecting potential blood-based biomarkers of cognitive frailty
Source: J Nutr Health Aging. 2025 Nov 24;30(1):100726. doi: 10.1016/j.jnha.2025.100726 (PMC12681723; doi:10.1016/j.jnha.2025.100726)
Supplement: Supplementary file 1 [file mmc1.pdf]

Supplementary materials

Method S1. Additional methodological details for RNA-seq data analysis..2
Table S1. Results of clinical data analysis..3
Table S2. Summary of RNA-seq data 6
Table S3. Results of metabolome analysis..9
Table S4. Relationships between candidate biomarkers, J-CHS scores, and cognitive function...........14
Figure S1. Outline of risk prediction model construction..15
Figure S2. Distribution of J-CHS score in this study...16
Figure S3. Result of RNA-seq...17
Figure S4. Correlation between candidate biomarkers J-CHS frailty score and cognitive function......18

Supplementary Method 1.

Additional methodological details for RNA-seq data analysis

The quality of the read sequences (fastq files) was assessed by using FastQC (ver. 0.11.7 and ver. 0.11.9). Low-quality reads (< Q20) and trimmed reads with adaptor sequences (shorter than 50 bp) were excluded by using Cutadapt (ver. 1.16). The remaining clean sequenced reads were mapped to a human reference genome (GRCh37) with STAR (ver. 2.5.2b). Read counts for each gene were calculated with the featureCounts program in the subread package (ver. 1.6.6) to generate expression levels. The read counts from each sample were combined into a count file, on which differential expression analysis was performed with edgeR (ver. 3.18.1). Genes with a threshold CPM (counts per million reads mapped) of > 1 in more than one-fourth of all sequenced samples were used for further analysis. The 'caclNormFactors' function in edgeR was used to obtain trimmed mean of M-values (TMM) normalization factors to account for library sizes. The 'exactTest' function in edgeR was applied to obtain differentially expressed genes (DEGs) between cognitive frailty and robust samples. DEGs were defined as genes with normalized transcripts per million (nTPM) ≥ 1 in total peripheral blood mononuclear cells (PBMCs) from the Human Protein Atlas database (https://www.proteinatlas.org), a |log2 (fold-change: FC)| > 1, and FDR < 0.05. FDR values were calculated by using the Benjamini–Hochberg method.

Supplementary Table 1. Results of clinical data analysis

Variable	Robust<br>Mean ± SD	Cognitive frailty<br>Mean ± SD	Odds ratio (95% CI)	*P*	FDR
Hemoglobin / Red cell distribution width ratio	1.01 ± 0.086 (n = 33)	0.90 ± 0.16 (n = 32)	0.30 (0.14–0.67)	3.20E-03	0.089
Albumin/globulin ratio	1.55 ± 0.26 (n = 33)	1.33 ± 0.23 (n = 32)	0.37 (0.18–0.74)	5.26E-03	0.089
Basophils (%)	0.66 ± 0.30 (n = 33)	0.44 ± 0.23 (n = 32)	0.41 (0.21–0.79)	8.24E-03	0.089
MCHC (%)	33.6 ± 1.13 (n = 33)	33 ± 0.84 (n = 32)	0.40 (0.21–0.77)	6.32E-03	0.089
Platelet distribution width (%)	10.6 ± 1.22 (n = 33)	11.7 ± 2.15 (n = 32)	2.33 (1.23–4.41)	9.24E-03	0.089
Red cell distribution width (%)	13.1 ± 0.73 (n = 33)	14.1 ± 1.63 (n = 32)	4.22 (1.46–12.2)	7.66E-03	0.089
Mean platelet volume (fL)	9.7 ± 0.73 (n = 33)	10.2 ± 1.07 (n = 32)	2.24 (1.19–4.22)	0.013	0.094
Years of education	12.9 ± 2.55 (n = 33)	11 ± 2.46 (n = 32)	0.42 (0.21–0.83)	0.013	0.094
Mean corpuscular hemoglobin (pg)	31.1 ± 1.36 (n = 33)	30.2 ± 2.31 (n = 32)	0.43 (0.21–0.85)	0.016	0.10
Total protein (g/dL)	7.03 ± 0.43 (n = 33)	7.27 ± 0.44 (n = 32)	2.03 (1.1–3.75)	0.024	0.14
Free thyroxine 4 (ng/dL)	1.01 ± 0.11 (n = 33)	1.07 ± 0.15 (n = 31)	1.89 (1.04–3.41)	0.036	0.19
SMI [InBody] (kg/m^2)	6.47 ± 0.82 (n = 33)	6.44 ± 1.17 (n = 30)	0.31 (.093–1.01)	0.052	0.24
Hemoglobin (g/dL)	13.1 ± 1.01 (n = 33)	12.5 ± 1.48 (n = 32)	0.54 (0.28–1.02)	0.057	0.24
Neutrophil count (×10^2/μL)	29.1 ± 7.85 (n = 33)	35.3 ± 11.5 (n = 32)	1.81 (0.99–3.31)	0.054	0.24
Cholinesterase (U/L)	337 ± 65.4 (n = 33)	304 ± 76.5 (n = 32)	0.59 (0.32–1.08)	0.089	0.34
Heart rate (bpm)	75.7 ± 11.0 (n = 33)	81.1 ± 15.8 (n = 32)	1.62 (0.91–2.87)	0.099	0.36
White blood cells (×10^2/μL)	50.9 ± 9.94 (n = 33)	57.7 ± 15.8 (n = 32)	1.63 (0.89–2.97)	0.11	0.39
Calcium (mg/dL)	9.31 ± 0.35 (n = 33)	9.36 ± 0.34 (n = 32)	1.53 (0.85–2.73)	0.16	0.45
25-Hydroxyvitamin D (ng/mL)	20.3 ± 7.05 (n = 32)	17.1 ± 4.87 (n = 32)	0.62 (0.32–1.19)	0.15	0.45
Bone-specific alkaline phosphatase (μg/L)	14.9 ± 6.61 (n = 32)	12.9 ± 4.11 (n = 32)	0.65 (0.36–1.18)	0.15	0.45

Parathyroid hormone, intact (pg/mL)	52.7 ± 19.2 (n = 33)	49.4 ± 20.9 (n = 32)	0.67 (0.39–1.17)	0.16	0.45
Direct bilirubin (mg/dL)	0.16 ± 0.068 (n = 33)	0.18 ± 0.073 (n = 32)	1.39 (0.82–2.36)	0.23	0.53
Neutrophils (%)	57.3 ± 9.81 (n = 33)	60.8 ± 9.06 (n = 32)	1.37 (0.79–2.37)	0.26	0.53
Albumin (g/dL)	4.24 ± 0.27 (n = 33)	4.11 ± 0.3 (n = 32)	0.70 (0.40–1.22)	0.20	0.53
Undercarboxylated osteocalcin (ng/mL)	5.08 ± 2.7 (n = 32)	4.35 ± 3.04 (n = 32)	0.72 (0.41–1.26)	0.26	0.53
NTx (nmol BCE/L)	16 ± 3.65 (n = 32)	14.7 ± 3.2 (n = 31)	0.71 (0.39–1.28)	0.25	0.53
Platelets (×10^4/μL)	22.8 ± 4.17 (n = 33)	23.3 ± 6.32 (n = 32)	1.41 (0.79–2.53)	0.25	0.53
Potassium (mEql/L)	4.18 ± 0.35 (n = 33)	4.09 ± 0.27 (n = 32)	0.70 (0.39–1.26)	0.24	0.53
Mean corpuscular volume (fL)	92.4 ± 2.99 (n = 33)	91.8 ± 6.27 (n = 32)	0.74 (0.43–1.28)	0.28	0.56
Monocytes (%)	6.88 ± 2.41 (n = 33)	6.56 ± 2.84 (n = 32)	0.74 (0.42–1.31)	0.30	0.56
eGFR (mL min^{-1} 1.73 m^{-2})	68.5 ± 14.1 (n = 32)	70.5 ± 16.9 (n = 28)	1.35 (0.75–2.44)	0.32	0.56
Hematocrit (%)	39.0 ± 3.2 (n = 31)	37.9 ± 4.0 (n = 32)	0.74 (0.42–1.32)	0.31	0.56
Triglyceride (mg/dL)	140 ± 78.2 (n = 31)	120 ± 78.4 (n = 27)	0.74 (0.41–1.33)	0.31	0.56
Total cholesterol (mg/dL)	203 ± 37.6 (n = 33)	192 ± 23.6 (n = 32)	0.77 (0.44–1.33)	0.35	0.59
Lymphocytes (%)	32.8 ± 8.65 (n = 33)	29.5 ± 7.54 (n = 32)	0.77 (0.44–1.35)	0.36	0.60
TRACP-5b (mU/dL)	432 ± 167 (n = 32)	414 ± 153 (n = 32)	0.8 (0.47–1.35)	0.41	0.66
Urea nitrogen (mg/dL)	17.5 ± 4.72 (n = 32)	16.7 ± 4.85 (n = 28)	0.81 (0.45–1.45)	0.48	0.71
C-reactive protein (mg/dL)	0.10 ± 0.23 (n = 33)	0.19 ± 0.34 (n = 32)	1.27 (0.68–2.38)	0.46	0.71
Creatinine (mg/dL)	0.71 ± 0.17 (n = 32)	0.69 ± 0.16 (n = 28)	0.77 (0.37–1.59)	0.48	0.71
Amylase (U/L)	79.9 ± 26.8 (n = 33)	74.2 ± 21.9 (n = 32)	0.84 (0.49–1.44)	0.52	0.71
High-density lipoprotein (mg/dL)	62.9 ± 15.7 (n = 31)	58.7 ± 9.04 (n = 27)	0.80 (0.42–1.53)	0.51	0.71
Chlorine (mEq/L)	105 ± 2.44 (n = 33)	106 ± 2.6 (n = 32)	1.19 (0.70–2.01)	0.53	0.71

Diastolic blood pressure (mm Hg)	78.8 ± 10.6 (n = 33)	78.7 ± 12.0 (n = 32)	1.20 (0.70–2.08)	0.51	0.71
Free thyroxine 3 (pg/mL)	2.58 ± 0.37 (n = 33)	2.59 ± 0.33 (n = 31)	1.18 (0.68–2.05)	0.55	0.73
Low-density lipoprotein (mg/dL)	112 ± 32.2 (n = 31)	108 ± 23.9 (n = 27)	0.85 (0.46–1.56)	0.60	0.74
YAM of femoral neck (%)	81.4 ± 14.4 (n = 33)	85.2 ± 13.4 (n = 31)	1.17 (0.66–2.07)	0.59	0.74
Indirect bilirubin (mg/dL)	0.52 ± 0.21 (n = 33)	0.48 ± 0.24 (n = 32)	0.87 (0.51–1.47)	0.60	0.74
Sodium (mEql/L)	142 ± 2.36 (n = 33)	142 ± 2.33 (n = 32)	1.11 (0.65–1.92)	0.70	0.84
YAM of lumbar spine (%)	92.7 ± 23.6 (n = 33)	95 ± 23.1 (n = 32)	0.90 (0.47–1.73)	0.76	0.89
Inorganic phosphorus (mg/dL)	3.53 ± 0.48 (n = 33)	3.52 ± 0.46 (n = 32)	1.09 (0.63–1.87)	0.77	0.89
Alanine aminotransferase (U/L)	19.3 ± 6.8 (n = 33)	18.5 ± 8.08 (n = 32)	0.94 (0.56–1.58)	0.81	0.89
Creatine kinase (U/L)	110 ± 48.4 (n = 33)	127 ± 104 (n = 32)	1.08 (0.62–1.88)	0.80	0.89
Total bilirubin (mg/dL)	0.68 ± 0.27 (n = 33)	0.64 ± 0.30 (n = 32)	0.94 (0.56–1.56)	0.80	0.89
Red blood cells ($\times 10^4/\mu L$)	422 ± 38.3 (n = 33)	415 ± 54.3 (n = 32)	0.96 (0.56–1.65)	0.88	0.94
Gamma-glutamyl transpeptidase (U/L)	26.2 ± 15.9 (n = 33)	26.2 ± 19.1 (n = 32)	1.01 (0.60–1.7)	0.97	0.99
Systolic blood pressure (mm Hg)	144 ± 16.3 (n = 33)	145 ± 18.8 (n = 32)	1.02 (0.61–1.69)	0.95	0.99
Eosinophils (%)	2.4 ± 2.07 (n = 33)	2.71 ± 2.84 (n = 32)	0.98 (0.56–1.70)	0.94	0.99
Aspartate aminotransferase (U/L)	21.5 ± 6.34 (n = 33)	22.4 ± 7.24 (n = 32)	1.01 (0.59–1.73)	0.99	0.99

Abbreviation: BCE = bone collagen equivalent; YAM = young adult mean; CI = confidence interval; eGFR = estimated glomerular filtration rate; MCHC = mean corpuscular hemoglobin concentration; NTx = cross-linked N-terminal telopeptide of type 1 collagen; SD = standard deviation; SMI = skeletal muscle index; TRACP-5b = Tartrate-resistant acid phosphatase-5b.

Supplementary Table 2. Summary of RNA-seq data

Subject ID	RIN	MMSE-J	MoCA-J	Input read	Unique map reads	% Unique map reads	Multi-map reads	% Multi-map reads	Q20
Cognitive frailty 1	6.5	26	20	23,610,136	18,825,504	79.73	3,221,163	13.64	98.76
Cognitive frailty 2	7.1	30	19	21,597,630	17,579,406	81.4	2,500,172	11.58	98.7
Cognitive frailty 3	6.7	28	19	20,615,243	17,283,193	83.84	1,908,795	9.26	98.51
Cognitive frailty 4	8.3	27	21	14,989,954	12,152,952	81.07	1,662,828	11.09	98.37
Cognitive frailty 5	6.4	29	23	28,457,220	23,159,565	81.38	2,989,645	10.51	98.4
Cognitive frailty 6	6.5	25	20	31,940,023	25,296,669	79.2	4,993,696	15.63	98.91
Cognitive frailty 7	7	27	21	25,142,750	20,334,621	80.88	2,870,259	11.42	98.63
Cognitive frailty 8	7.8	26	25	25,728,838	18,379,257	71.43	5,588,831	21.72	98.38
Cognitive frailty 9	7.9	28	19	19,091,880	14,589,717	76.42	1,791,112	9.38	96.98
Cognitive frailty 10	7.4	28	24	16,455,874	12,058,455	73.28	2,002,320	12.17	96.06
Cognitive frailty 11	7.8	25	25	23,868,982	19,275,466	80.76	2,847,054	11.93	98.43
Cognitive frailty 12	8	27	25	25,782,699	19,988,765	77.53	3,790,512	14.7	98.11
Cognitive frailty 13	7.8	25	22	18,321,954	14,685,473	80.15	2,417,094	13.19	98.79
Cognitive frailty 14	7.1	29	20	20,337,524	16,442,415	80.85	2,630,750	12.94	98.84
Cognitive frailty 15	7.3	28	24	23,346,035	18,992,610	81.35	2,629,675	11.26	98.7
Cognitive frailty 16	6.9	26	25	23,131,878	18,558,985	80.23	3,212,250	13.89	99.01
Cognitive frailty 17	7.2	26	20	22,724,003	18,368,960	80.84	2,956,192	13.01	98.89
Cognitive frailty 18	6.9	29	25	21,107,169	16,313,377	77.29	3,107,748	14.72	98.54
Cognitive frailty 19	7.9	26	21	16,601,829	11,675,327	70.33	2,892,305	17.42	96.62
Cognitive frailty 20	7.4	29	25	34,496,270	29,342,678	85.06	2,999,827	8.7	98.98
Cognitive frailty 21	7.2	26	24	30,766,760	26,063,097	84.71	3,726,596	12.11	99.4
Cognitive frailty 22	8.4	26	22	36,256,771	30,420,605	83.9	3,898,070	10.75	99.27
Cognitive frailty 23	8.2	28	24	34,812,215	26,075,160	74.9	6,533,732	18.77	98.93
Cognitive frailty 24	8.1	26	17	22,896,535	18,138,833	79.22	4,099,654	17.91	99.46
Cognitive frailty 25	7.1	24	24	31,081,782	27,277,714	87.76	2,928,286	9.42	99.49
Cognitive frailty 26	7.4	25	22	34,530,221	28,936,013	83.8	4,621,827	13.38	99.64
Cognitive frailty 27	7.5	30	23	35,770,028	27,183,453	76	7,534,877	21.06	99.54
Cognitive frailty 28	8	30	25	17,406,258	14,012,851	80.5	2,242,536	12.88	98.27
Cognitive frailty 29	7.4	25	21	33,827,691	27,912,868	82.51	4,957,493	14.66	99.45

Cognitive frailty 30	7.4	25	20	22,792,397	20,361,361	89.33	1,130,085	4.96	99.01
Cognitive frailty 31	6.9	30	23	26,533,500	23,562,741	88.8	1,701,146	6.41	99.25
Cognitive frailty 32	7	25	17	28,318,605	22,983,945	81.16	3,099,433	10.94	98.37
Cognitive frailty 33	7.4	30	24	26,501,633	22,266,557	84.02	2,402,052	9.06	98.63
Cognitive frailty 34	6.5	26	21	44,741,380	32,072,262	71.68	9,525,661	21.29	98.92
Cognitive frailty 35	6.7	24	13	23,704,943	18,112,580	76.41	3,623,884	15.29	98.46
Cognitive frailty 36	6.1	25	16	20,879,073	15,894,196	76.13	3,587,499	17.18	98.63
Cognitive frailty 37	8.4	30	22	30,265,332	23,067,840	76.22	5,723,256	18.91	99.15
Cognitive frailty 38	6.4	28	22	17,353,735	14,838,139	85.5	1,281,440	7.38	98.59
Cognitive frailty 39	7.8	25	16	18,930,731	15,698,025	82.92	2,009,602	10.62	98.9
Cognitive frailty 40	7.7	25	18	33,225,241	29,416,425	88.54	2,300,521	6.92	99.32
Cognitive frailty 41	7.5	25	22	22,049,080	18,957,398	85.98	1,673,747	7.59	99.05
Cognitive frailty 42	6.2	30	19	32,345,564	27,183,439	84.04	3,345,165	10.34	99.13
Cognitive frailty 43	7.8	27	22	23,165,300	18,964,046	81.86	2,635,950	11.38	98.62
Robust 1	6.9	28	26	19,415,412	16,032,032	82.57	1,971,162	10.15	98.55
Robust 2	6.3	30	26	31,147,799	25,280,541	81.16	4,635,615	14.88	99.29
Robust 3	7.3	30	29	25,371,494	19,098,600	75.28	3,116,491	12.28	96.76
Robust 4	7.6	30	28	59,992,047	49,457,416	82.44	7,721,530	12.87	99.29
Robust 5	7.1	29	26	14,881,161	12,349,793	82.99	1,336,532	8.98	98.23
Robust 6	7.6	30	28	26,853,477	21,443,128	79.85	3,503,922	13.05	98.76
Robust 7	6.7	30	30	23,114,076	18,895,602	81.75	2,562,679	11.09	98.71
Robust 8	8.2	29	28	27,528,547	22,898,131	83.18	3,075,377	11.17	99.23
Robust 9	7.4	30	26	35,273,486	28,061,448	79.55	4,930,322	13.98	98.78
Robust 10	7.9	29	27	26,325,956	21,783,040	82.74	2,988,429	11.35	99.08
Robust 11	7.2	30	27	34,298,969	28,382,064	82.75	3,620,103	10.55	99.02
Robust 12	7.2	30	26	20,076,444	17,492,693	87.13	2,069,108	10.31	99.56
Robust 13	7.3	30	27	30,224,885	22,523,771	74.52	6,892,825	22.81	99.56
Robust 14	8.1	29	28	36,584,381	32,100,891	87.74	3,407,289	9.31	99.55
Robust 15	8.4	29	26	57,143,524	48,892,387	85.56	6,469,925	11.32	99.46
Robust 16	6.9	30	28	39,327,966	32,397,951	82.38	5,757,350	14.64	99.63
Robust 17	7.7	29	27	41,042,130	36,450,781	88.81	3,507,615	8.55	99.61

Robust 18	7.9	30	26	27,406,357	21,083,791	76.93	4,368,843	15.94	98.3
Robust 19	7.2	28	26	27,336,506	23,011,340	84.18	3,597,956	13.16	99.52
Robust 20	6.4	30	28	26,044,803	23,504,015	90.24	1,083,596	4.16	99.26
Robust 21	7.7	30	26	30,341,821	26,797,992	88.32	1,805,137	5.95	98.93
Robust 22	6.7	30	28	42,560,919	35,410,792	83.2	4,572,490	10.74	99.19
Robust 23	7.3	29	27	26,476,387	22,129,504	83.58	2,712,946	10.25	98.87
Robust 24	6.8	28	29	22,668,576	18,929,247	83.5	2,421,017	10.68	98.82
Robust 25	7.2	29	27	25,864,928	22,730,805	87.88	1,641,352	6.35	98.95
Robust 26	6.9	29	27	22,420,102	19,934,640	88.91	1,325,607	5.91	99.17
Robust 27	6.8	30	26	36,398,389	30,443,220	83.64	4,989,017	13.71	99.58
Robust 28	7.3	29	27	44,227,920	39,165,179	88.55	2,322,039	5.25	98.89
Robust 29	7.7	29	27	29,796,975	26,473,065	88.84	1,591,631	5.34	99.02
Robust 30	6.9	30	26	22,628,195	19,572,199	86.49	1,747,322	7.72	98.92
Robust 31	7.8	30	26	24,231,839	19,708,096	81.33	3,597,256	14.85	99.06
Robust 32	7.2	30	27	27,202,783	23,391,735	85.99	2,757,557	10.14	99.1
Robust 33	7.2	30	26	20,973,245	17,309,932	82.53	2,327,006	11.1	98.9
Robust 34	7.4	29	28	35,586,608	31,322,583	88.02	3,300,999	9.28	99.54
Robust 35	8.4	30	28	20,589,387	14,627,567	71.04	5,131,689	24.92	99.05
Robust 36	6.7	29	26	61,677,624	51,202,161	83.02	7,047,718	11.43	99.12
Robust 37	7	28	29	24,121,195	20,360,463	84.41	2,267,236	9.4	99.01
Robust 38	6.5	30	27	22,228,008	19,108,044	85.96	1,684,760	7.58	98.67
Robust 39	6.3	30	28	31,442,410	27,645,269	87.92	1,965,426	6.25	99.06
Robust 40	6.7	30	26	24,533,337	21,729,013	88.57	1,258,999	5.13	98.92
Robust 41	6.6	30	28	18,258,048	16,151,358	88.46	764,606	4.19	98.6
Robust 42	6.5	30	27	28,951,158	23,690,344	81.83	3,212,307	11.1	98.55
Robust 43	6.7	28	27	26,801,260	18,113,077	67.58	6,438,167	24.02	98.17
Robust 44	7.9	30	26	21,398,686	15,503,114	72.45	4,324,050	20.21	98.54

Abbreviation: MMSE = Mini-Mental State Examination; MoCA-J = Japanese version of the Montreal Cognitive Assessment; Q20, read quality score of 20; RNA-seq = RNA sequencing; RIN = RNA integrity number.

Supplementary Table 3. Results of metabolome analysis

Metabolite (uM)	Robust<br>Mean ± SD	Cognitive frailty<br>Mean ± SD	Odds ratio (95% CI)	*P*	FDR
**Myristic acid**	0.042 ± 0.0096	0.027 ± 0.010	0.18 (0.074–0.43)	1.16E-04	**0.015**
**4-Methylpyrazole**	0.025 ± 0.0051	0.020 ± 0.0037	0.24 (0.10–0.56)	9.72E-04	**0.047**
**Nicotinamide**	0.031 ± 0.010	0.022 ± 0.0085	0.30 (0.14–0.62)	1.28E-03	**0.047**
**γ-Butyrobetaine**	0.024 ± 0.0045	0.021 ± 0.0051	0.33 (0.17–0.65)	1.46E-03	**0.047**
Imidazolelactic acid	0.029 ± 0.0094	0.021 ± 0.0079	0.24 (0.095–0.59)	2.05E-03	0.053
Lactic acid	1720 ± 575	2120 ± 793	2.22 (1.07–4.62)	0.030	0.31
Homoarginine	0.027 ± 0.011	0.020 ± 0.010	0.43 (0.20–0.90)	0.025	0.31
γ-glutamyl ornithine	0.019 ± 0.0048	0.022 ± 0.0056	1.96 (1.05–3.69)	0.036	0.31
Hypoxanthine	2.07 ± 1.08	2.75 ± 1.46	2.03 (1.08–3.82)	0.028	0.31
Histidine	85.3 ± 15.3	75.7 ± 11.60	0.48 (0.25–0.95)	0.036	0.31
Glutathione disulfide	0.30 ± 0.14	0.24 ± 0.12	0.51 (0.27–0.97)	0.039	0.31
Gluconolactone	0.023 ± 0.0041	0.026 ± 0.050	1.97 (1.07–3.61)	0.029	0.31
5-Amino-3,4-dihydro-2H-pyrrole-2-carboxylic acid	0.025 ± 0.016	0.021 ± 0.013	0.41 (0.18–0.95)	0.037	0.31
1-Methyladenosine	0.021 ± 0.0040	0.020 ± 0.0042	0.47 (0.24–0.90)	0.023	0.31
N1-Acetylspermidine	0.017 ± 0.0045	0.022 ± 0.0074	2.33 (1.18–4.61)	0.015	0.31
Asymmetric dimethylarginine	0.019 ± 0.0021	0.021 ± 0.0038	2.07 (1.06–4.07)	0.034	0.31
Valine	261 ± 58.1	232 ± 49.30	0.52 (0.28–0.98)	0.042	0.32
cis-Aconitic acid	9.17 ± 1.73	10.6 ± 2.54	2.03 (1.01–4.05)	0.046	0.33
Tyrosine	90.3 ± 24.2	80.9 ± 20.90	0.61 (0.35–1.08)	0.091	0.38
Sarcosine	2.67 ± 1.01	2.22 ± 0.58	0.58 (0.32–1.04)	0.069	0.38
Threonic acid	0.025 ± 0.0081	0.022 ± 0.0056	0.58 (0.32–1.05)	0.074	0.38
Aminoacetone	0.022 ± 0.0043	0.02 ± 0.0036	0.61 (0.35–1.07)	0.085	0.38
Alloisoleucine	0.024 ± 0.0068	0.022 ± 0.0061	0.55 (0.28–1.09)	0.089	0.38

Uric acid	0.017 ± 0.0049	0.020 ± 0.0045	1.67 (0.93–2.99)	0.087	0.38
Propionylcarnitine	0.024 ± 0.0073	0.021 ± 0.0082	0.64 (0.37–1.09)	0.10	0.38
p-Cresol sulfate	0.020 ± 0.016	0.028 ± 0.021	1.64 (0.92–2.93)	0.093	0.38
Pipecolic acid	0.039 ± 0.072	0.022 ± 0.014	0.14 (0.015–1.22)	0.075	0.38
Uridine	13.9 ± 2.98	14.8 ± 2.19	1.62 (0.92–2.87)	0.10	0.38
Trigonelline	0.031 ± 0.038	0.015 ± 0.012	0.37 (0.12–1.16)	0.09	0.38
Butyrylcarnitine	0.031 ± 0.017	0.023 ± 0.012	0.54 (0.29–1.02)	0.06	0.38
Glycerol	0.017 ± 0.0068	0.021 ± 0.010	1.7 (0.94–3.07)	0.08	0.38
Lauric acid	0.024 ± 0.0026	0.023 ± 0.0031	0.63 (0.35–1.1)	0.09	0.38
Creatinine	63.3 ± 16.4	60.6 ± 14.10	0.56 (0.29–1.09)	0.09	0.38
Leucine	142 ± 44	122 ± 31.30	0.6 (0.33–1.08)	0.09	0.38
Cysteine glutathione disulfide	0.015 ± 0.0058	0.018 ± 0.0083	1.68 (0.90–3.12)	0.10	0.38
Taurine	0.023 ± 0.0058	0.020 ± 0.0065	0.60 (0.31–1.13)	0.11	0.40
Carnitine	0.021 ± 0.0031	0.020 ± 0.0036	0.63 (0.36–1.12)	0.11	0.40
Methionine sulfoxide	0.028 ± 0.012	0.023 ± 0.0075	0.60 (0.32–1.15)	0.13	0.42
Cysteine	0.018 ± 0.0040	0.021 ± 0.0048	1.63 (0.87–3.07)	0.13	0.42
Ethanolamine	0.019 ± 0.0040	0.021 ± 0.0035	1.57 (0.87–2.83)	0.13	0.43
Ornithine	58.1 ± 14.7	66.3 ± 18.90	1.49 (0.87–2.57)	0.15	0.46
γ-Carboxyglutamic acid	0.021 ± 0.0034	0.020 ± 0.0046	0.64 (0.35–1.17)	0.15	0.46
Paraxanthine	0.024 ± 0.013	0.018 ± 0.012	0.66 (0.37–1.17)	0.16	0.46
Betaine	62.7 ± 16.0	58.6 ± 11.30	0.68 (0.40–1.17)	0.17	0.49
Phosphorylcholine	0.022 ± 0.0065	0.020 ± 0.0071	0.69 (0.40–1.18)	0.17	0.49
Citrulline	38.7 ± 10.0	35.3 ± 8.94	0.68 (0.39–1.21)	0.19	0.49
Creatine	53.5 ± 27.5	58.8 ± 23.30	1.44 (0.83–2.48)	0.19	0.49
Diethanolamine	0.021 ± 0.0064	0.019 ± 0.0066	0.65 (0.34–1.24)	0.19	0.49
H-Asp(Gly-OH)-OH	0.023 ± 0.0083	0.021 ± 0.0057	0.69 (0.39–1.22)	0.20	0.49
Caffeine	0.028 ± 0.019	0.018 ± 0.018	0.65 (0.34–1.25)	0.20	0.49

Hypotaurine	0.017 ± 0.0062	0.019 ± 0.0074	1.46 (0.82–2.57)	0.20	0.49
Symmetric dimethylarginine	0.019 ± 0.0040	0.021 ± 0.0041	1.52 (0.82–2.8)	0.20	0.49
Proline	187.0 ± 64.6	173 ± 65.40	0.71 (0.42–1.22)	0.21	0.51
1-Methylnicotinamide	0.028 ± 0.012	0.023 ± 0.014	0.68 (0.37–1.26)	0.22	0.52
3-Hydroxybutyric acid	62.2 ± 50.2	80.9 ± 76.10	1.4 (0.82–2.39)	0.23	0.52
Homocitrulline	0.019 ± 0.0095	0.023 ± 0.011	1.48 (0.77–2.83)	0.24	0.55
5-Oxoproline	0.019 ± 0.0042	0.019 ± 0.0040	1.35 (0.79–2.32)	0.27	0.57
Glutamic acid γ-methyl ester	0.018 ± 0.0057	0.017 ± 0.0061	0.74 (0.42–1.27)	0.27	0.57
3-Aminoisobutyric acid	0.016 ± 0.013	0.021 ± 0.015	1.37 (0.78–2.4)	0.27	0.57
γ-Glutamyl arginine divalent	0.020 ± 0.0045	0.018 ± 0.0049	0.73 (0.43–1.26)	0.26	0.57
Choline	22.4 ± 3.99	21.9 ± 4.98	0.72 (0.40–1.29)	0.27	0.57
Urea	0.023 ± 0.0048	0.022 ± 0.0059	0.74 (0.44–1.26)	0.27	0.57
Trimethylamine N-oxide	0.025 ± 0.031	0.019 ± 0.012	0.70 (0.36–1.36)	0.29	0.58
3-Aminobutyric acid	0.021 ± 0.0044	0.022 ± 0.0055	1.38 (0.76–2.48)	0.29	0.58
Galacturonic acid	0.020 ± 0.0059	0.019 ± 0.0060	0.73 (0.40–1.33)	0.30	0.58
Thiaproline	0.017 ± 0.0068	0.020 ± 0.0088	1.39 (0.75–2.58)	0.30	0.58
4-Oxopyrrolidine-2-carboxylic acid	0.022 ± 0.0053	0.021 ± 0.0050	0.74 (0.42–1.31)	0.31	0.59
Methionine	17.9 ± 5.78	16.4 ± 4.24	0.76 (0.44–1.31)	0.32	0.60
Lysine	258.0 ± 50.9	243 ± 46.30	0.77 (0.46–1.31)	0.34	0.63
γ-Glutamyl alanine	0.020 ± 0.0069	0.019 ± 0.0070	0.76 (0.43–1.34)	0.35	0.63
Arginine	98.4 ± 26.6	87.7 ± 28.10	0.77 (0.45–1.32)	0.34	0.63
Stachydrine	0.026 ± 0.040	0.019 ± 0.024	0.75 (0.42–1.37)	0.35	0.63
Alanine	390 ± 93.7	370.0 ± 96.0	0.77 (0.43–1.37)	0.37	0.65
Mucic acid	0.026 ± 0.0086	0.024 ± 0.0069	0.79 (0.45–1.36)	0.39	0.67
Glutamine	746 ± 88.6	711 ± 100.00	0.78 (0.44–1.38)	0.40	0.67
N6-Methyllysine	0.018 ± 0.015	0.020 ± 0.015	1.25 (0.71–2.22)	0.44	0.71
N-Acetylgalactosamine	0.014 ± 0.0038	0.016 ± 0.0039	1.27 (0.70–2.29)	0.44	0.71

Glycine	236 ± 68.3	244 ± 62.00	1.25 (0.72–2.18)	0.42	0.71
2-Oxoglutaric acid	14.6 ± 2.72	14.2 ± 3.08	0.80 (0.45–1.42)	0.44	0.71
Isoleucine	82.3 ± 29.4	76.3 ± 22.20	0.81 (0.48–1.38)	0.44	0.71
Ethanolamine phosphate	0.02 ± 0.0063	0.022 ± 0.0088	1.25 (0.69–2.26)	0.46	0.72
Octanoylcarnitine	0.024 ± 0.013	0.021 ± 0.012	0.81 (0.46–1.43)	0.48	0.73
γ-Glutamyl isoleucine	0.021 ± 0.0053	0.020 ± 0.0062	0.83 (0.49–1.4)	0.48	0.73
Isocitric acid	13.0 ± 2.82	14.1 ± 3.64	1.23 (0.70–2.15)	0.48	0.73
γ-Glutamyl valine	0.018 ± 0.0041	0.020 ± 0.0058	1.22 (0.69–2.17)	0.49	0.73
Guanidinoacetic acid	0.022 ± 0.0063	0.020 ± 0.0074	0.81 (0.45–1.47)	0.49	0.73
Pyruvic acid	157 ± 46.6	168 ± 49.60	1.22 (0.67–2.23)	0.51	0.74
Glutamic acid	69.8 ± 23.8	67.6 ± 23.70	0.83 (0.48–1.44)	0.51	0.74
Asparagine	48.1 ± 10.2	44.8 ± 10.60	0.83 (0.49–1.43)	0.51	0.74
γ-Glutamyl lysine divalent	0.021 ± 0.0038	0.021 ± 0.0046	0.84 (0.50–1.42)	0.52	0.74
Hydroxyproline	10.8 ± 5.52	10.5 ± 5.00	0.84 (0.49–1.44)	0.53	0.74
3-Indoxylsulfuric acid	0.020 ± 0.016	0.023 ± 0.012	1.17 (0.70–1.95)	0.56	0.78
Threonine	128 ± 41	132 ± 33.10	1.16 (0.69–1.95)	0.57	0.78
N6-Acetyllysine	0.020 ± 0.0051	0.021 ± 0.0053	1.16 (0.68–1.97)	0.58	0.79
2-Hydroxybutyric acid	39.3 ± 12.5	44.5 ± 24.50	1.2 (0.62–2.34)	0.59	0.79
Phenylalanine	70.5 ± 12.7	68.6 ± 16.90	0.86 (0.51–1.48)	0.59	0.79
Glycerophosphocholine	0.020 ± 0.0058	0.019 ± 0.0048	0.86 (0.48–1.53)	0.60	0.79
γ-Glutamylglutamine	0.020 ± 0.0053	0.019 ± 0.0057	0.88 (0.51–1.5)	0.63	0.80
Pyrocatechol sulfate	0.022 ± 0.012	0.019 ± 0.020	0.88 (0.51–1.51)	0.63	0.80
Citric acid	187 ± 36.3	194 ± 37.50	1.15 (0.65–2.02)	0.64	0.80
L-Aspartic acid	9.3 ± 3.83	9.13 ± 4.11	0.88 (0.51–1.51)	0.64	0.80
Serine	125 ± 29.9	120 ± 24.70	0.87 (0.5–1.52)	0.63	0.80
Isethionic acid	0.019 ± 0.0048	0.019 ± 0.0057	0.90 (0.53–1.52)	0.69	0.84
Gluconic acid	6.99 ± 1.53	7.4 ± 1.98	1.12 (0.65–1.93)	0.68	0.84

Cystathionine	0.020 ± 0.0076	0.022 ± 0.014	0.89 (0.49–1.6)	0.69	0.84
Tryptophan	47.7 ± 10.7	47.5 ± 9.09	1.11 (0.66–1.89)	0.70	0.84
4-Methyl-2-oxovaleric acid	0.019 ± 0.0048	0.020 ± 0.0043	1.11 (0.63–1.97)	0.71	0.84
Guanidinosuccinic acid	0.024 ± 0.011	0.024 ± 0.015	0.90 (0.53–1.53)	0.71	0.84
Terephthalic acid	0.020 ± 0.0028	0.020 ± 0.0034	0.92 (0.53–1.59)	0.76	0.90
S-Methylcysteine	0.020 ± 0.011	0.020 ± 0.0065	0.93 (0.56–1.56)	0.79	0.91
N-Acetylalanine	0.019 ± 0.0038	0.020 ± 0.0054	1.09 (0.59–2.01)	0.78	0.91
2-Oxoisovaleric acid	12.1 ± 2.25	12.6 ± 3.20	1.09 (0.58–2.05)	0.79	0.91
O-Acetylcarnitine	0.020 ± 0.0049	0.021 ± 0.0048	1.05 (0.61–1.82)	0.85	0.96
Adenosine diphosphate	5.85 ± 2.11	5.82 ± 2.92	0.96 (0.53–1.73)	0.88	0.98
2-Aminoisobutyric acid	0.020 ± 0.0057	0.019 ± 0.0053	1.04 (0.61–1.77)	0.88	0.98
Malic acid	7.22 ± 2.21	7.68 ± 2.43	0.99 (0.56–1.76)	0.97	0.99
Kynurenine	0.020 ± 0.0056	0.021 ± 0.010	1.01 (0.55–1.85)	0.98	0.99
1-Methylhistidine	0.019 ± 0.010	0.018 ± 0.014	0.98 (0.56–1.71)	0.94	0.99
2-Hydroxyvaleric acid	0.019 ± 0.019	0.021 ± 0.026	0.98 (0.56–1.72)	0.94	0.99
3-Hydroxy-2-methyl-4-pyrone	0.022 ± 0.0045	0.022 ± 0.046	1.03 (0.60–1.77)	0.90	0.99
3-Hydroxyproline	0.020 ± 0.0055	0.020 ± 0.0060	1.02 (0.60–1.74)	0.94	0.99
5-Hydroxylysine	0.020 ± 0.010	0.021 ± 0.0068	0.98 (0.58–1.66)	0.94	0.99
Hippuric acid	0.023 ± 0.021	0.024 ± 0.021	1.03 (0.59–1.77)	0.93	0.99
Iminodiacetic acid	0.020 ± 0.0041	0.019 ± 0.032	0.98 (0.58–1.65)	0.94	0.99
N6,N6,N6-Trimethy-L-lysine	0.021 ± 0.0082	0.022 ± 0.012	1.01 (0.60–1.71)	0.98	0.99
β-Alanine	3.44 ± 1.77	3.35 ± 2.19	0.99 (0.59–1.65)	0.96	0.99
Ascorbate 2-sulfate	0.020 ± 0.0045	0.020 ± 0.0073	0.99 (0.56–1.72)	0.96	0.99
N,N-Dimethylglycine	4.69 ± 1.53	4.79 ± 1.64	1.00 (0.60–1.66)	1.00	1.00

Abbreviation: CI = confidence interval; FDR = false discovery rate; SD = standard deviation.
Associations significant at FDR < 0.05 are shown in bold font.

Supplementary Table 4. Relationships between candidate biomarkers, J-CHS scores and cognitive function

Analysis	Candidate biomarker	Scores	Effect size (95% CI)	*P*	FDR	No of associations
ELISA		J-CHS				
	GDF15	**Shrinking**	6.70 (1.37–32.8)*	0.019	**0.0033**	
		Exhaustion	0.84 (0.38–1.85)*	0.66	0.66	
		Low activity	1.60 (0.85–3.03)*	0.15	0.16	3
		**Slowness**	3.48 (1.45–8.38)*	5.32E-03	**0.014**	
		**Weakness**	2.19 (1.08–4.44)*	0.029	**0.044**	
		MMSE-J/MoCA-J				
		**MMSE-J**	-0.76 (-1.21–0.30)†	0.0014	**0.0062**	2
		**MoCA-J**	-0.95 (-1.78– -0.12)†	0.0026	**0.0038**	
Metabolome		**J-CHS**				
	Myristic acid	**Shrinking**	8.39E-03 (1.21E-04–0.58)*	0.027	**0.038**	
		**Exhaustion**	0.18 (0.052–0.61)*	6.19E-03	**0.014**	
		**Low activity**	0.32 (0.16–0.63)*	9.52E-04	**5.00E-3**	**5**
		**Slowness**	0.18 (0.088–0.38)*	4.55E-06	**9.45E-05**	
		**Weakness**	0.41 (0.19–0.91)*	0.027	**0.038**	
		MMSE-J/MoCA-J				
		**MMSE-J**	0.62 (0.22–1.03)†	0.0029	**0.0085**	2
		**MoCA-J**	1.15 (0.44–1.85)†	0.0018	**0.0062**	
		J-CHS				
	Nicotinamide	Shrinking	0.28 (0.071–1.07)*	0.063	0.074	
		Exhaustion	0.43 (0.19–1.00)*	0.050	0.062	
		**Low activity**	0.47 (0.25–0.86)*	0.015	**0.028**	2
		**Slowness**	0.25 (0.12–0.50)*	1.06E-04	**1.12E-03**	
		Weakness	0.45 (0.20–1.06)*	0.067	0.074	
		MMSE-J/MoCA-J				
		**MMSE-J**	0.57 (0.15–1.00)†	0.0084	**0.018**	2
		**MoCA-J**	1.41 (0.68–2.14)†	2.36E-04	**1.65E-03**	

Abbreviation: CI = confidence interval, FDR = false discovery rate;

J-CHS = Japanese version of the Cardiovascular Health Study, MMSE-J= Mini-mental State Examination-Japanese, MoCA-J= the Japanese version of Montreal Cognitive Assessment

*: Logistic regression adjusted age, sex, BMI, †: Linear regression adjusted age, sex and Body mass index

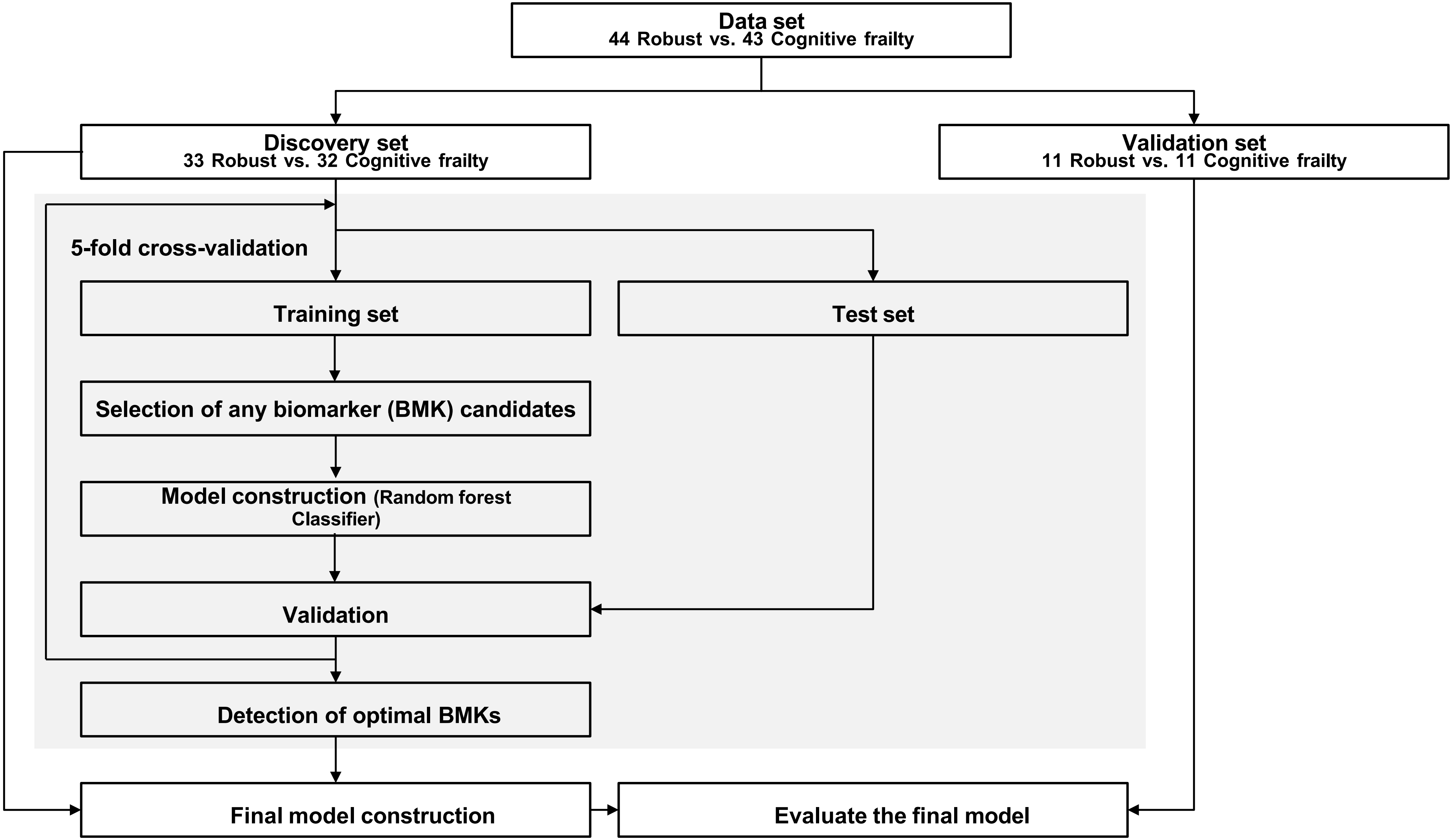

Supplementary Figure1. Outline of risk prediction model construction.

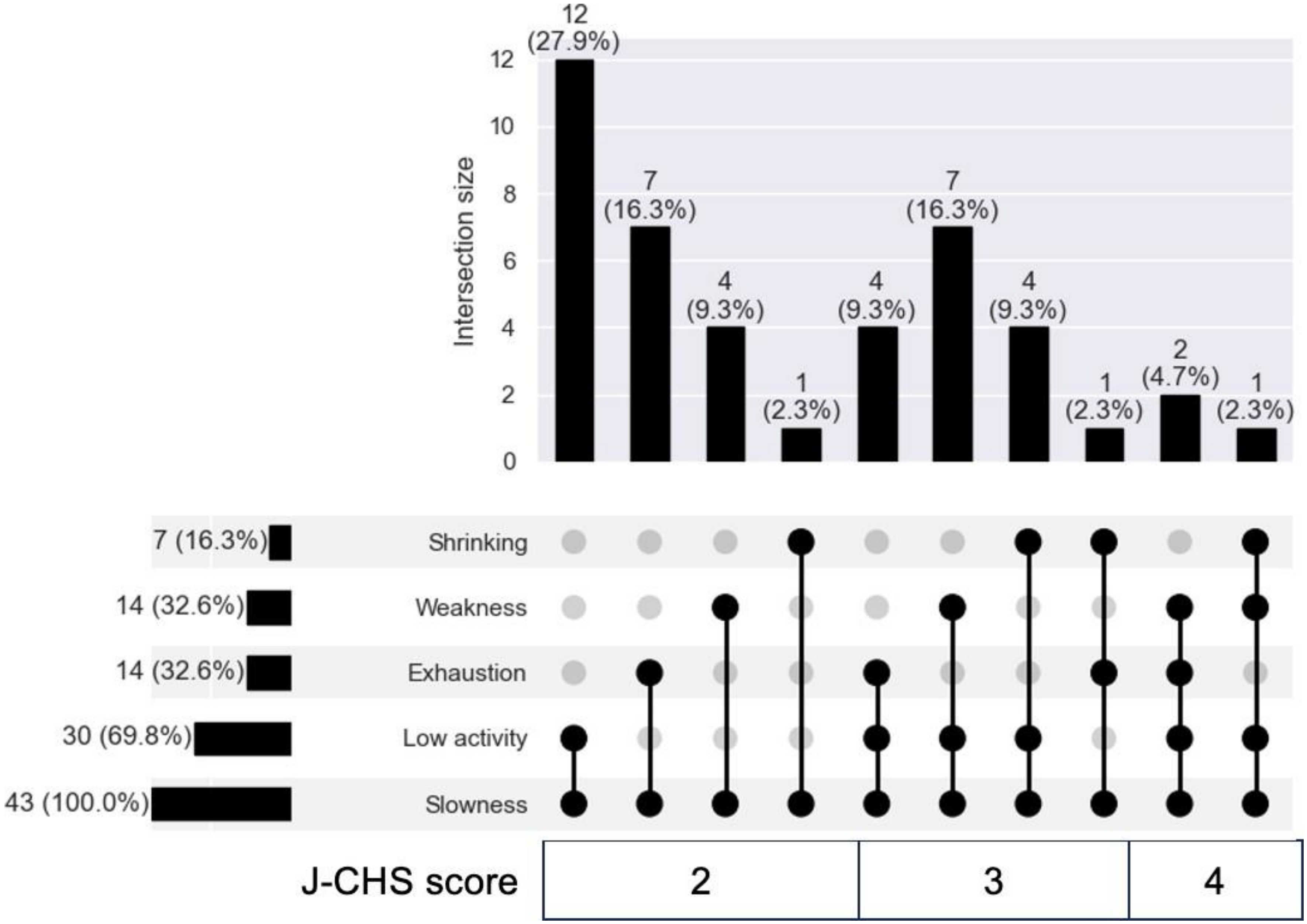

Supplementary Figure 2. Distribution of J-CHS score in this study.
The distribution of J-CHS score among 43 cognitive frailty older adults. J-CHS = Cardiovascular Health Study in Japanese.

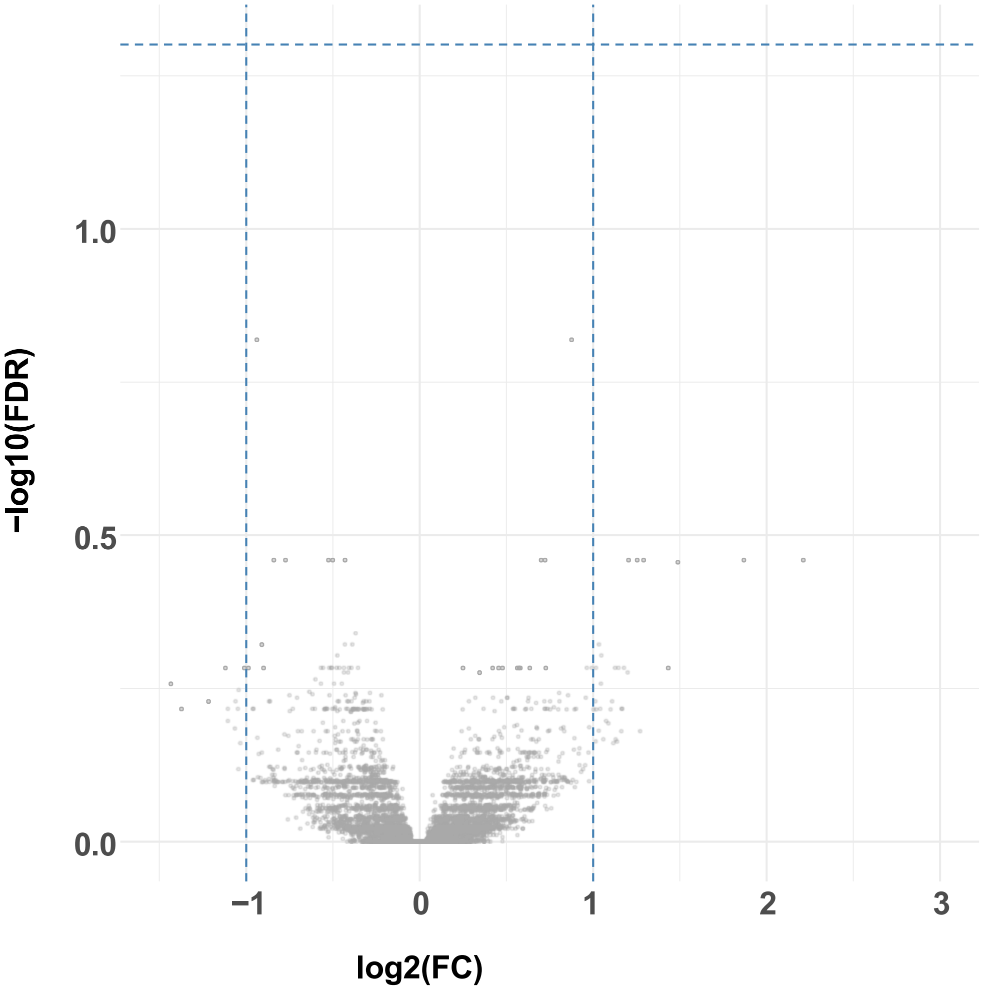

Supplementary Figure3. Result of RNA-seq

Differentially expressed genes (DEG) detection by RNA-seq. Each point represents a DEG. There were no significant DEGs (false discovery rate <0.05, $|\log_2(\text{fold change})[FC]| \geq 1$, and normalized transcriptome per million ≥ 1 in total peripheral blood mononuclear cells from the Human Protein Atlas (https://www.proteinatlas.org)

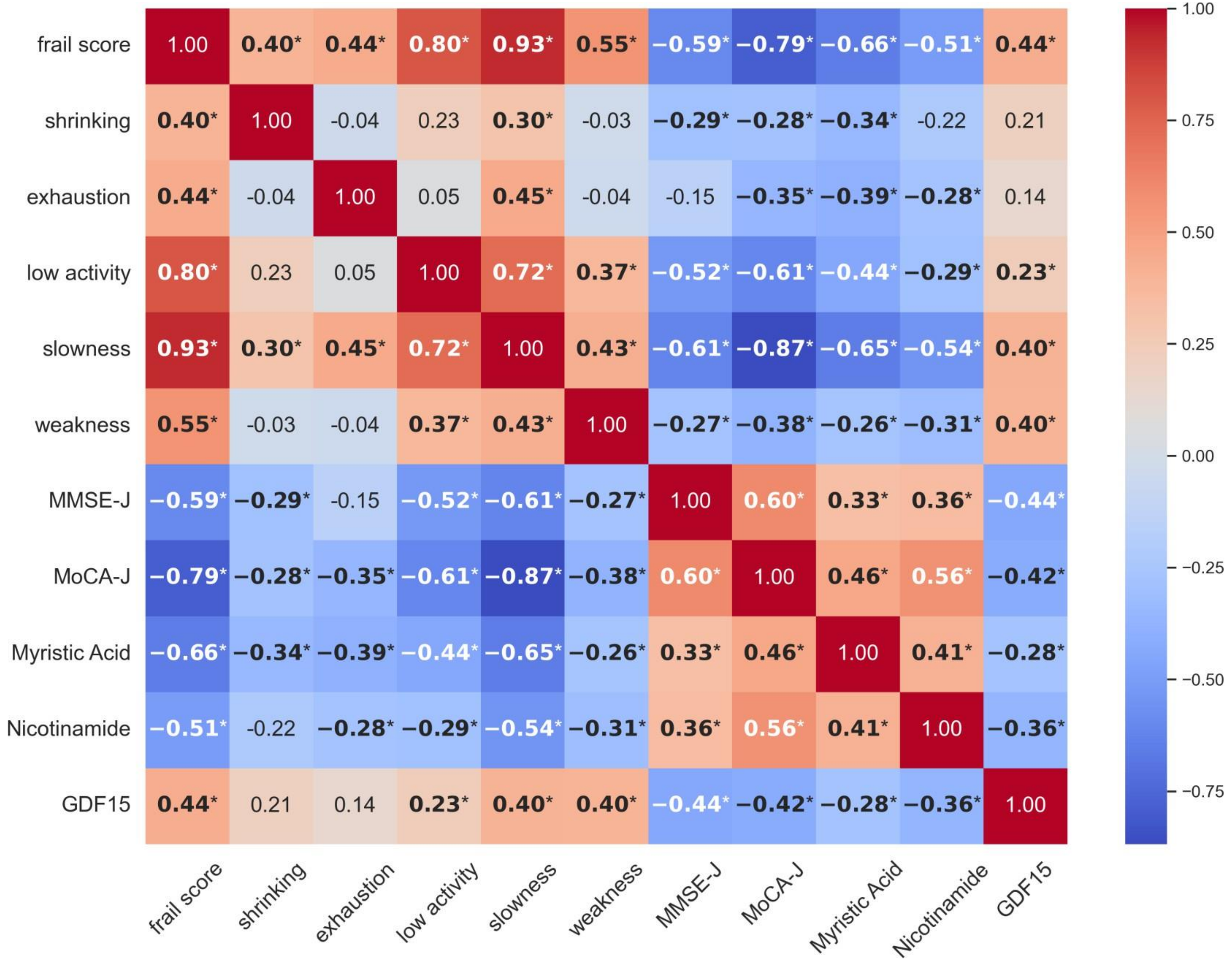

Supplementary Figure 4. Correlation between candidate biomarkers, J-CHS frailty score and cognitive function

Correlation was estimated using Spearman's correlation coefficient. *: $P < 0.05$.
